# Letter from Dr. Flint, Paris

**Published:** 1854-10

**Authors:** 


### BUFFALO MEDICAL JOURNAL

AND

### MONTHLY REVIEW.

**VOL. 10.** OCTOBER, 1854. **NO. 5.**

### ORIGINAL COMMUNICATIONS.

ART. I.—*Editorial Correspondence.* Letter from Prof. FLINT.

PARIS, June 18th, 1854.

DEAR DOCTOR: My previous letters have related, for the most part, to the hospitals of Paris. I propose in this letter to give you some account of the *école de médicine*.

The *école de médicine,* the seat of the *faculty of medicine*, in the Academy of Paris, is situated in the place *d'école de médicine,* on a street bearing the same title, on the left bank of the Seine, in what is generally known as the *Latin quarter* of Paris. The present edifice was commenced in 1769, the corner-stone being laid by Louis XV, and was inaugurated in 1776. I quote a description of its architecture from the Paris Guide by GALIGNANI. "The front toward the street is 198 feet in length; the lateral wings are connected by a portico formed of a double range of coupled Doric columns, interrupted by an arched entrance leading into a rectangular court, and surmounted by a bas-relief representing Louis XV, accompanied by Wisdom and Beneficence granting privileges to the school of surgery, and the Genius of the Arts presenting to the King a plan of the building. The court is 66 by 96 feet. At the bottom is a portico of six Corinthian columns, of large proportions, resting on steps and surmounted by a pediment. The bas-relief of the tympanum represents Theory and Practice joining hands on an altar. The inner frieze of this portico bears medallions, with the portraits in bas-relief, of PITARD, DE LA PEYRONNIE, PARE, MARECHAL, and PETIT. The amphitheatre to which it leads will contain 1400 students. It is a hemicycle, lit by a sky-light, and contains a monochrome fresco by GIBELIN, dated 1775, illustrative of the utility of the medical science."

In addition to the fresco ornaments just mentioned, the amphitheatre contains several busts and a splendid painting intended to commemorate the substitution of the ligature for the hot iron in arresting hemorrhage, underneath which is the following inscription:—*Ils étanchent le sang consacré à la defense de la patrié. La bienfaisance du soverain hate leur progrés, et recompense leur zéle. Ils tiennent des Dieux les principes qu'ils nous ont transmis.*"

On the opposite part of the ampitheatre is inscribed the following motto: —"*Ad cœdes hominum prisca ampitheatra patebant. Ut longum discant vivere nostra patent.*"

The walls have a dingy color, no effort being made to diminish the appearance of antiquity by the use of whitewash or paint. A niche contains a kind of pulpit, elevated four or five feet from the floor, for the lecturer; or he occupies a chair upon the floor behind a table covered with green baize. All the lectures that I have heard have been delivered literally from chairs, i. e. the professors sitting.

The seats for students consist of plain board benches without railing or backs. As regards convenience and comfort for the lecturer and his auditory, the arrangements are inferior to those of any medical lecture-room in the United States that I have seen. Moreover, there is but a single amphitheatre, or lecture-room in the building. The lectures are given in succession, in the same room, including those on anatomy and chemistry.

Four lectures are given daily during the winter and summer; at present as follows: two from 10½ to 12½ A. M., and two from 3 to 5 P. M. The courses now going on are on Medical Natural Philosophy, by GAVARRET; Materia Medica, GRISOLLE; Hygiene, BOUCHARDAT; Surgical Pathology, CLOQUET and BROCHA; Medical Pathology, REQUIN; Pathological Anatomy, Legal Medicine, ADELON. During the winter, courses are given on several branches which do not enter into the summer session, viz: Anatomy, Physiology, and Chemistry.

Attendance at these courses is not obligatory upon the medical student pursuing his studies for a degree. No tickets are issued, and admission is free to any one. Hence, the different courses are attended by different persons, and the size of the auditory differs according to the subject, and the popularity of the lecturer. At the close of one lecture a considerable portion of the class leave the ampitheatre, and as the hour is about to expire persons are coming in who desire to attend the lecture which is to follow. As there is little or no interval between the ending of one lecture and the beginning of another, this change of auditory gives rise to considerable noise and confusion. There is no applause when the professor enters the amphitheatre, and very rarely any demonstration of approbation when the lecture is ended. The students generally sit with their hats on, and a large proportion take notes. Whenever I have been present the demeanor of the class has been respectful and decorous, except that more or less come in after the lecture has been commenced, and leave during its delivery.

The *faculty of medicine* consists of 26 persons, appointed by government since the accession of Napoleon III. They receive a fixed salary from government, varying from four hundred to two thousand dollars. Their emolument is thus entirely independent of the number of students. It is left optional with the student to avail himself of their lectures or not. It is not a requisition for graduation that he shall have attended any of them. He is obliged to pursue the study of medicine for four years before he is entitled to a degree; and the fact of this requisition having been complied with is determined by means of quarterly inscriptions. He must inscribe his name at the bureau of the faculty every three months, having previously acquired the diploma of a bachelor of sciences. At the end of the first year he is examined in chemistry, natural history and botany. The second examination takes place at the expiration of the third year. He is then examined in anatomy and physiology. At the end of the fourth year, provided all his inscriptions have been duly made, he is examined in external and internal pathology, hygiene, medical jurisprudence, pharmacy, materia medica, therapeutics and midwifery. He is also subjected to a practical examination at the bed-side, two hospital cases being selected, of which he is required to give the diagnosis, prognosis and treatment. He is also obliged to write and have printed a thesis, the subject of which is optional; and before taking the degree of Doctor of Medicine he must have served a year in a hospital.

The examinations of medical students are public. I chanced to be in the museum of Orfila a few days since while three examining boards were in session. The examiners were clothed in their official costume, consisting of caps with red crowns, gold bands and tassels; gowns of red silk faced with black, and bands tipped with ermine; or black gowns and red collars. The examinations were conducted in a familiar, colloquial manner. They did not appear to be severe, but, in general, so long as I stood among the crowd of spectators, they were sustained but indifferently by the candidates. Official notice of the result of the examination in each individual instance is conspicuously placed on the advertising board within the court of the *école.* I noticed in this announcement that a few had been found perfect; others satisfactory; some passable, and several had acquitted themselves very poorly.

The expenses incident to a medical education here consist of a fee of from six to ten dollars for each of the sixteen inscriptions; a fee of six dollars paid at each of the five examinations; twelve dollars for an examination of the thesis; twenty dollars for the diploma, and from forty to fifty dollars for printing the thesis. The total amount is from two hundred and twenty to two hundred and sixty dollars. This is exclusive of private courses, requiring fees, which the student may see fit to take during he four years; and also exclusive of the expenses of dissection, which, however, are moderate.*

The expenses of medical education have reference only to those who aim at a medical degree. To medical men of all countries, and to those who wish to study any branch as a matter of science or accomplishment, not with a view to practice, the lectures and all other facilities are open without charge.

The *école de médicine* contains a library of 30,000 volumes. This is open daily, excepting Sundays and Thursdays, from the hour of 11 till 4. The second story of the left lateral wing is devoted to this purpose. The library room is provided with tables, comfortable chairs and ink stands, and from thirty to fifty persons may here generally be seen reading or writing, each attending to his own business, no audible conversation being permitted.

* One can form but an imperfect idea of the number of medical students in Paris by the size of the classes at the different lectures, at the *ècole de mèdicine;* still less by the attendance at any one of the hospitals. The only way to obtain correct information on this point is to ascertain the number of inscriptions. The number in 1852 was 1,437. A great many foreign countries are represented among the students collected here—Germans, Swedes, Russians, Turks, Greeks, etc. It is rare to meet with students or practitioners from Great Britain. The number of Americans is always considerable, but the larger proportion have graduated before coming to Paris. It is not easy for an American to determine with much exactness how many of his medical countrymen are here, because each having his particular objects and preferences, they are distributed in groups at the different hospitals, and private courses. From what I can learn there were probably not far from a hundred here during the winter. At the present time the number is diminished by one third at least.

The respect for order and propriety of deportment so conspicuous in all the public places in Paris, is sufficient guaranty against infractions of this rule.

The front of the edifice, and a portion of the right lateral wing contain the *Orfila museum*, so called in commemoration of the services rendered by Orfila to science and medical education. The space allotted to the museum embraces a hall extending the entire front of the edifice, with a gallery on all sides, and two rooms in the right wing, about sixty feet in length. The front hall is devoted to Anatomy, human and comparative. A marble statue of Cuvier stands in this hall, represented in a sitting posture, examining a fossil. At the bottom of the staircase leading to the museum, is a colossal statue, in plaster, of Bichat, represented counting the pulsations of the heart in a child standing nude before him.

The central space of the large hall into which one first enters, is filled with skeletons, complete and united, of a large number of animals—the horse, cow, lion, bear, dog, kangaroo, hyena, cougar, wild boar, ant-eater, etc., etc. The smaller specimens are contained in glass cases affixed to the walls, on every side. These specimens are systematically arranged in the following classes:—osteology, arthrology, aponeurology, myology, neurology, ovology; lymphatics and lacteals, organs of the circulation, of digestion, of respiration, of secretion, elementary tissues. Each of these classes is illustated by a great number and variety of beautiful specimens. I will notice some of these groups briefly, in order that you may form a general idea of the extent of the museum. To describe in detail would require too much space.

The department of human osteology embraces several skeletons united, a series of detached bones, collections of the vertebral bones united, pelvis, and a great number of skulls, and plaster casts of crania. The division of comparative osteology, in addition to the skeletons already referred to, includes several hundred skeletons, and crania of inferior animals.

The myological group consists of dried preparations, in many of which the volume of the muscles appears but little diminished, finely colored, the latter prepared by M. Lucquet, and artificial models by Auzoux. These preparations, natural and artificial, embrace a great variety of inferior animals as well as man.

In the class neurology, the brain and nerves are exhibited in a great variety of modes by means of wet and dried preparations, and models in wax. The superficial nerves of the lower extremity are beautifully shown on the integument turned inside out, like the finger of a glove, dried and varnished, the volume of the limb being preserved. The spinal cord and nerves in a great variety of inferior animals are displayed in jars, suspended in a dark purple fluid. The organs of sense are illustrated by a great number of specimens. I counted sixty-five small jars containing wet preparations of the various structures composing the eye, eighty-nine dried preparations of different parts of the same organ, and seventy-eight artificial models—in all two hundred and thirty-two distinct pieces.

Under the head of ovology, an oil painting first meets the eye, representing a female, of the size of life, in parturition, the head just emerging from the vulva. Next comes a series of wax preparations illustrating the development of the ovum, and the different stages of gestation, from the entrance of the ovum within the uterus to the full period, after the observations of W. Erdl, Professor in the University of Munich. Over the series, on a metallic plate is inscribed the following:—"*Evolutio ovi embryonis que humani quam observarit, et per* Paulum Teiller *in cera effigendam curavit.* Dr. M. Erdl, *medicinæ professor p. o. monarchii*, 1845." This class also embraces a great number of fœtuses of all ages.

The lymphatics and lacteals are finely displayed by mercurial injections. Among the preparations are several of the mesentery and portions of the intestines of the horse, with the lacteal vessels injected.

The organs of the circulation include numerous specimens of the human heart in alcohol; a great number injected and dried, representing every age; different portions and sections of the organ; the heart and lungs united, and the chest with all the organs *in situ.* Preparations of arteries and veins injected are very numerous, and brilliantly colored. Among the latter I noticed several, showing the relations of the blood-vessels of the thigh to the membranes, made with great care and very beautiful.

The respiratory organs consist of the vocal organs in different animals, and a number of dried preparations of the larynx in man, the veins in general being displayed. These parts are also represented by several wax models. The varieties of conformation in the breathing apparatus in the different genera of animals are illustrated, and the human lungs of fœtal life, and at different ages after birth.

Arranged under the head of organs of secretion, are preparations of the liver and kidneys, minutely injected with size; of the mammary gland injected with mercury, and a formidable array of the male organs of generation. The penis is presented in every variety of size, form and color, and describing all sorts of parabolic curves. I had the curiosity to count the dried preparations of the latter organ; the number was one hundred and five.

Two rooms are appropriated to natural history, materia medica, and surgical instruments. Here are large collections of shells, insects, and the different genera of inferior animals. Articles of the materia medica are exhibited in jars and in open glass vases. The anatomical and surgical instruments make a fine display. Among them is exhibited the case used for the autopsy of Napoleon. In this part of the museum, amongst other objects pertaining to natural history, is a model in wax of the famous dwarf *Bebe*, twenty inches in height.

From this very cursory sketch it will be justly inferred that the Orfila museum is worthy of the *école de médicine* of Paris. I have never seen a collection, so large and splendid, of objects illustrative of human and comparative anatomy, natural history, and materia medica. I have not, however, as yet compared it with other museums in Europe, especially with the Hunterian museum of London, which I have been told is superior. Collections illustrating the same departments of science are to be found at the *Jardin des plants*, in addition to the extensive botanical and mineralogical cabinets at the latter place. I have not yet found time to examine these• The cabinet of comparative anatomy at the *Jardin des plants* is said to be the richest in existence. For this prominence it is mainly indebted to the labors of the great CUVIER. The geological collection also is immense, consisting of upwards of 200,000 specimens, all systematically arranged to exhibit the successive gradations of animal life from man to the simplest form of manifestation.

Neither the Orfila museum, nor the cabinets at the *Jàrdin des plants*, contain illustrations of disease. The *Musée Dupuytren* is devoted to morbid anatomy. This museum is contained in a building erected for this purpose, situated but a few rods from the *école de médicine.* It contains a large number of diseased and fractured bones, wet and dried specimens, and artificial models. They are all nicely prepared and systematically arranged in glass cases. In magnitude and richness, however, it does not correspond with the Orfila museum. Another less extensive pathological museum is connected with the ampitheatre of anatomy at *Clamart,* situated in another part of the city.

The *école pratique d'anatomie* is a branch of the *école de médicine*, and is situated but a short distance, adjoining the *Musée Dupuytren.* Here, and at Clamart, just mentioned, dissections are performed. To these two places the bodies of those who die at the hospitals, and are unclaimed by friends, are transferred. The number of subjects received annually exceeds 4,000. The supply is always abundant, and the expense to the student very moderate, a fee of six dollars securing all that he requires for a season.

Lectures are also given at both places by *Aggrégés*, a corps of subordinate professors, who are awaiting vacancies to be promoted to regular chairs. This corps embraces those most eminent for talent and acquirement irrespective of the regular professors in the *école de médicine*, and as they have the latter position to win, they are the active working men among the distinguished teachers. Their courses are generally gratuitous, but sometimes a small fee is required. These lectures are exclusive of the great number and variety of private courses which are given by persons at their own rooms or dispensaries. For the latter an inscription with a fee are sometimes demanded, and in other instances there is no charge.*

The lectures at the *école de médicine*, the *école pratique*, and the private courses; the clinical visits, lectures, and conferences at the hospitals; the dissections and the museums, constitute the sources and means of medical instruction in this metropolis. In this connection I might naturally propose to myse.f the question in what particulars, and to what extent, will the American medical student find in these sources and means of instruction advantages which are not equally available at home. To this question I have given considerable thought since I came to Paris, and I shall continue to keep it in view during the remainder of my sojourn here, with the intention of making it a subject of discussion hereafter. For the present I shall waive any thing like a discussion of it. That there are certain facilities for pursuits pertaining to medical science to be found here which do not exist in the same degree with us, must be admitted. But in admitting this, I am not willing for a moment to concede that the fact involves, of necessity, any inferiority in the position of the American medical profession, individually or collectively. Attainment, skill and just claim to eminence, in medical, as in other pursuits, Providence has ordained to depend mainly on mental ability and industry. If the sciences of life and disease may in some respects be prosecuted under more favorable auspices in Europe, every thing necessary to the highest point of acquisition is attainable at home, and, as an American, I am reluctant to believe that the American mind need fail to accomplish, even under some difficulties, as much as the successful intellectual laborer in other countries. If it be true that the standard of medical education is higher here than with us; if in the ranks of the profession the

---

* It is a fact well known here, that much the larger proportion of the amount of fees paid for the private courses, and to *internes* for clinical instruction at the hospitals, comes from foreigners, and especially from the American students and practitioners who visit Paris for medical improvement.

number is greater of those conspicuous for valuable services rendered to medicine, and if the medical literature of France is richer and more copious than ours, it behooves us to inquire into the reasons for such a disparaging contrast, and then endeavor to do away with the grounds on which it rests. I believe that, at the present moment, our country can boast of members of the medical profession as able and industrious as those of any other country. We have teachers, writers and practitioners who would not suffer by comparison, in every respect, with the most distinguished on this side of the Atlantic. It rests with ourselves to take a national position, as regards the science of medicine, in keeping with the political importance of our republic. In deciding respecting the expediency of advising young men to come to Paris to pursue medical studies, there are considerations to be taken into account other than those which concern the opportunities for improvement which are here offered. Giving due weight to the latter, as well as the former, I do not hesitate to express the opinion that the American student, in the majority of instances, will do best to remain at home. I shall content myself with expressing this opinion, adjourning, as I have said, the discussion of the subject to a future time.

The appointments to official places, excepting the professorships, as is well known, are determined by the "*concours*." The *concours* consists of a public competition for vacant offices by the candidates, and is intended to constitute a fair test of the superior talents and attainments of the successful aspirants. Certain subjects are given to the competitors for impromptu discussion, orally, and in writing. They must also write and defend publicly a thesis on a given subject.

They who acquit themselves best, are adjudged by the Faculty entitled to the places competed for. Until the accession of the present Emperor to the throne of France, this was the system pursued in making all the appointments. It is still retained with respect to hospital physicians and surgeons, the *internes* and *externes*, and *aggrégés*. The professors, however, are appointed by the Emperor. The system of *concours* undoubtedly has many recommendations. Its influence on the profession must be good. It is, moreover, based on a principle of equity. It provides against incompetent incumbents. If, however, common report be correct, experience shows that it is not secure against partiality, and, as a test of qualifications, it is by no means infallible. The permanency of the appointments to professorships, and the complete independence of the professors as respects their classes, although just when the offices are considered as the reward of past services, are of doubtful expediency with reference to the best interests of education

It is, however, wisely arranged that attendance on the lectures by the professors is not obligatory; and the medical student is at liberty to avail himself of the instruction of those who have not reached the *ultimatum* of official position, and have therefore greater inducements to continued exertion.

I will try to write two or three more letters while I remain here if you think it worth while to do so. I will give an account of *St. Louis* hospital, of the Academy of Medicine and other Societies, of the Medical Journals, and of the customs of practitioners, &c. A. F.

